# Right frontal anxiolytic-sensitive EEG ‘theta’ rhythm in the stop-signal task is a theory-based anxiety disorder biomarker

**DOI:** 10.1038/s41598-021-99374-x

**Published:** 2021-10-05

**Authors:** Shabah M. Shadli, Lynne C. Ando, Julia McIntosh, Veema Lodhia, Bruce R. Russell, Ian J. Kirk, Paul Glue, Neil McNaughton

**Affiliations:** 1 Department of Psychology, University of Otago, PO Box 56, Dunedin, 9054 New Zealand; 2 School of Pharmacy, University of Otago, Dunedin, New Zealand; 3 Department of Psychological Medicine, University of Otago, Dunedin, New Zealand; 4 Department of Psychology, University of Auckland, Auckland, New Zealand

**Keywords:** Psychology, Biomarkers, Translational research

## Abstract

Psychiatric diagnoses currently rely on a patient’s presenting symptoms or signs, lacking much-needed theory-based biomarkers. Our neuropsychological theory of anxiety, recently supported by human imaging, is founded on a longstanding, reliable, rodent ‘theta’ brain rhythm model of human clinical anxiolytic drug action. We have now developed a human scalp EEG homolog—goal-conflict-specific rhythmicity (GCSR), i.e., EEG rhythmicity specific to a balanced conflict between goals (e.g., approach-avoidance). Critically, GCSR is consistently reduced by different classes of anxiolytic drug and correlates with clinically-relevant trait anxiety scores (STAI-T). Here we show elevated GCSR in student volunteers divided, after testing, on their STAI-T scores into low, medium, and high (typical of clinical anxiety) groups. We then tested anxiety disorder patients (meeting diagnostic criteria) and similar controls recruited separately from the community. The patient group had higher average GCSR than their controls—with a mixture of high and low GCSR that varied with, but cut across, conventional disorder diagnosis. Consequently, GCSR scores should provide the first theoretically-based biomarker that could help diagnose, and so redefine, a psychiatric disorder.

## Introduction

Anxiety disorders are a serious problem. They are currently the most prevalent psychiatric diseases^1,2^, the sixth highest cause of years of life lived with disability^3^, and may cause more than 5% of all suicide mortality^3^. They tend to start early in life^4,5^ and cause chronic impairment^6^.

Anxiety disorders are also hard to treat. Pharmacological treatment is weakly targeted. A variety of drugs is prescribed, often serially, in a wide range of cases; with poor predictive success^5^ and causing many problems even when they are effective^7^. In most clinical trials with pharmacological and psychological treatment, the response rate is only 50–60% and remission 25–35%^8^. While cognitive-behavioral therapy can be used as a general first line psychotherapeutic treatment for a range of anxiety disorders, there is no similar basis for choosing the drug, or even class of drug, that is appropriate for a particular patient. Along with others, we think that “Patients with mental disorders deserve better”^9^.

A key problem for drug targeting is weak diagnostic criteria for “anxiety disorders”. Disorders of defensive reactions currently receive many specific diagnoses within two main systems: The World Health Organization International Classification of Diseases, now in its 10th Edition (ICD-10)^10^; and the American Psychiatric Association’s Diagnostic and Statistical Manual, now in its 5th edition (DSM-5)^11^. This categorization of specific anxiety disorders is constantly evolving^12^, with both systems subdividing mental disorders using lists of surface-level clinical signs/symptoms (analogous to fever or breathlessness in systemic medicine) in contrast to defined syndromes (e.g. COVID-19) based on fundamental biological causes (e.g. SARS-CoV-2). Even the newer DSM-5^11^ has no unique objective identifier for any psychiatric disorder. Additionally, neither ICD-10 nor DSM-5 allows for comorbidity. They aim for a single diagnosis; however patients can have mixed presentations that fit multiple diagnoses; and comorbidity of anxiety with, for example, depression results in poorer prognosis and treatment response^8^. Additionally, “anxiety” and “panic” symptoms could co-occur with fundamental pathology limited to the control of either only anxiety or only panic systems^13^.

What is needed is biomarkers with strong theoretical foundations as emphasized in the Research Domain Criteria of the National Institute of Mental Health (https://www.nimh.nih.gov/research/research-funded-by-nimh/rdoc/index.shtml)^14^. We lack even a generally accepted definition of anxiety^15^; and have little understanding of “anxiety disorder” etiology and pathophysiology. This must change if we are to effectively diagnose and treat anxiety disorders^4^. Drug discovery is hampered by the lack of an adequate neuropsychological account of the mechanisms underlying anxiety disorders^12^; as are clinical trials of drugs that could treat anxiety, which often have very high relative rates of placebo response^4^. So, before embarking on clinical trials, many pharmaceutical companies and funding bodies increasingly seek a specific biological target relevant to the disease^4^.

Preclinical neuropsychology indicates a solution. We have developed, over several decades^16–20^, a highly detailed two dimensional (direction; distance)^21^ theory of defensive reactions, their neuropsychology, and their disorders. This theory has also been used as the basis of the Reinforcement Sensitivity Theory of human personality^22^. The fundamental axiom of our theory^16,17^ is that anxiolytic drugs act on, and so define, a Behavioral Inhibition System (BIS). The BIS is an “anxiety” system^20,21^ with a key role in processing goal conflict (e.g. approach-avoidance conflict). We define anxiolytic drugs as those acting at GABA_A_ or 5HT_1A_ receptors or voltage-gated calcium channels, which as a class can improve general anxiety symptoms in some cases but, unlike panicolytics such as fluoxetine, do not improve panic, phobia, depression or obsession^23^.

The septo-hippocampal system is a core element of the BIS^20,21^. Rhythmic EEG activity in the 4–12 Hz (‘theta’) range controls the BIS as a whole and may be a specific anxiety process biomarker^20^. (We place theta in quote marks below, since 4–12 Hz is referred to as ‘theta’ in the rodent literature and so spans the conventional human EEG theta and alpha bands^24^). Importantly, BIS function depends on ‘theta’ that, in the rat hippocampus, predicts human clinical anxiolytic action with, so far, no false positives (even with sedatives) or negatives (even with drugs ineffective in panic or depression)^25^. Repair of lost hippocampal ‘theta’ repairs behavioral dysfunction^26^; and hippocampal ‘theta’ mediates anxiolytic action on behavioral inhibition in approach-avoidance (and other goal) conflict^27^.

From the goal conflict aspect of the BIS theory^15,20^, and from our ‘theta’ anxiolytic model^25^, we have developed a human scalp EEG anxiety-process biomarker. Hippocampal ‘theta’ itself cannot be recorded from the scalp; but we showed in rats that, during risk assessment, it becomes phase locked with anterior frontal and cingulate cortex^28^. So, for our previous human translation work,

our primary hypothesis, driven by the BIS theory, was that conflict should be a source of avoidance, separate from simple aversion. To assess the unique influence of conflict on neural activation and behavior, we manipulated dollar gains and losses in a simple choice task. We predicted that when the potential amounts of gain and loss for a response were equal (generating approach–avoidance conflict), this should increase right frontal^29–34^ theta spectral power more than either net gain (greater approach tendency) or net loss (greater avoidance tendency). … [As predicted,] in the first half of the pre-response period, theta power peaked in CONFLICT trials at the right frontal site F8^35^, pp. 396, 398–399.

We then attempted to confirm the role in conflict of right frontal areas in general and F8 in particular, using the Stop Signal Task^36^ (SST). In the SST, the participant normally makes a left or right mouse click (‘go’) in response to a left (< =) or right (= >) arrow. However, if a tone is presented, they must withhold responding (‘stop’). Variation of the delay of a ‘stop’ signal can result in approach (‘go’, short delay), avoidance (‘stop’, long delay), or a conflict between the two when the theoretically independent^36^ approach and avoidance tendencies are balanced and there is about 50% correct stopping.

To test for stop-specific increases in EEG spectral power within the 4- to 12-Hz range at Fz, F4, and F8 in trials with intermediate delays, as compared to those with either short or long delays. If goal conflict was detected, we predicted that it would be processed as an aversive signal, and so individuals with high trait anxiety and/or neuroticism should show higher goal-conflict-specific EEG power^37^, p. 486.

As predicted, we found a right-frontal goal-conflict-specific EEG rhythmicity (GCSR) that was: (a) in the rodent hippocampal ‘theta’ frequency range (4–12 Hz); (b) positively correlated with neuroticism and trait anxiety^37^; and, (c) reduced by all key (non-panicolytic) classes of anxiolytic drugs^38–40^. Note that ‘theta’ recorded from the human dorsal hippocampus in a virtual reality model of a standard rodent test appears to have a power band of 5–11 Hz with a peak at 8 Hz^41^; similar to both rat dorsal hippocampus ‘theta’ and our anxiety process biomarker.

Here, we used a version of the SST that we had previously optimized for right frontal EEG (F8) GCSR detection^39^ to ask if the anxiety process for which GCSR is a biomarker is linked to some form of anxiety disorder. Answering this question is difficult because the theory assumes that high ‘theta’ will result in symptoms that match across a range of current diagnoses; and that symptoms and syndromes will be poorly matched^13^. That is, ‘theta’ should be high in some but not all cases of currently diagnosed anxiety disorder and should have similar effects across current nominal (symptom-based) diagnoses. Further, both the frequency and the amplitude of ‘theta’ can vary across situations, and across individuals within a situation; and it is not clear how far either or both contribute to anxiety disorders in people.

We have taken a form of cross-validation approach by first looking for, and refining the measurement of, an at least marginal increase in the predicted GCSR signal across a heterogenous pool of students, divided into groups with low, medium, and high (clinical level) trait “anxiety”. Although we were not using machine learning, this can be viewed as a ‘training sample’. Then, using methods based on this initial analysis, we tested a separately recruited patient group (pooled across anxiety diagnoses) against community controls; and then dissected the result obtained with the pooled patient group for its relationship with specific current diagnoses. These latter two analyses can be viewed as being performed on a ‘testing sample’.

We have used two anchors for this work. Our primary anchor, used in the ‘training sample’, is the Trait scale of the Spielberger State-Trait Anxiety Inventory (STAI-T)^42^. This is by no means a pure measure of a single anxiety trait nor a measure purely of anxiety but has a good relation with clinical anxiety disorders at the high end and, importantly, is designed to give a range of scores through the healthy population. It is also not subject to experimenter (or interviewer) bias. Our second anchor, used in the ‘testing sample’, has been receipt by a participant of any DSM anxiety disorder diagnosis. Given that our goal is to challenge the DSM nosology, this may seem odd. But, while we believe the specific categories within DSM (or ICD) need improvement and anchoring to biology, we believe that we can take a pool of people with *any* of the DSM diagnoses as being one that should have some with the required dysfunction compared to healthy controls. So, we will test the clinical importance of our biomarker using a pool of DSM diagnoses; and then later ask how much (or how little) our biomarker distinguishes between diagnoses or is a common feature within any one diagnosis.

**Figure 1:**
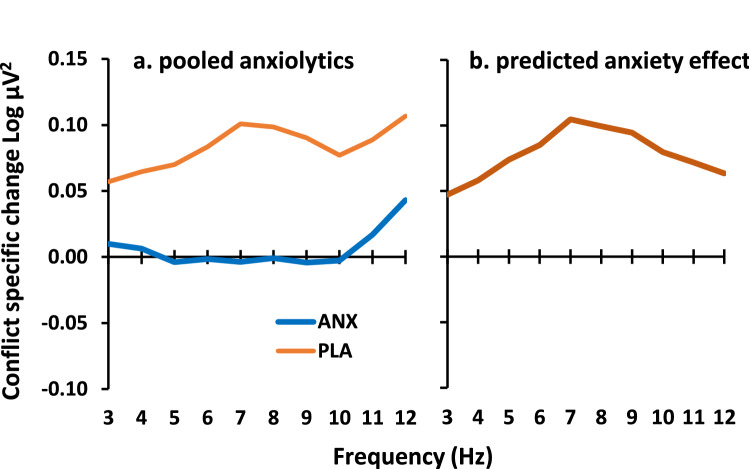
Predicted GCSR difference in high anxiety cases. **(**a) Average GCSR over three classes of anxiolytic drug (ANX, N = 26) compared with placebo (PLA, N = 8). Data are from the same participants as we reported previously^39^, but with different analysis parameters matching those of the current paper and with 3-point smoothing of Fig. 4. (**b**) The difference curve between these two groups as an estimate of expected anxiety-related power change predicted for high trait anxiety and for clinically diagnosed anxiety relative to their respective controls (c.f. Fig. 4).

To estimate the expected effect of high anxiety we determined the inverse of the effects of buspirone, triazolam and pregabalin from our previously reported drug data^39^. We averaged across the three classes of anxiolytic drug and carried out all other calculations as for the simple difference data reported below in Fig. 4. As shown in Fig. 1a, the drugs reduced GCSR in the range 5–10 Hz. To predict the approximate effect of high versus low trait anxiety, we subtracted these drug values from the placebo values. This resulted in a difference curve with a peak in the region of 7–8 Hz and a largely symmetrical fall-off on either side to 3 and 12 Hz (Fig. 1b). This curve is consistent with the power variation seen in human dorsal hippocampus during a test designed to replicate rodent ‘theta’ generation^41^. We predicted that the difference curves for both high trait anxiety students versus low and, separately, for patients versus healthy community volunteers would follow the same form. In terms of the approach taken below, this re-analysis of published data can be viewed as the first of two ‘training’ runs for the extraction of GCSR in the clinical ‘testing’ case.

## Results

**Figure 2:**
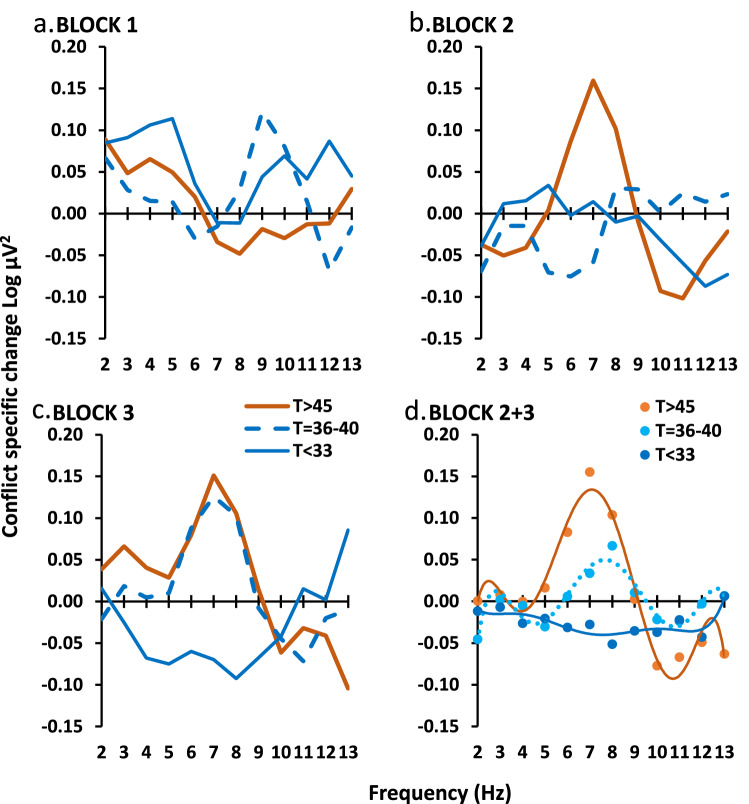
Variation of GCSR with blocks in the student sample. Groups are matched for gender and selected on STAI-T value (see main text). (**a**) There was no significant group difference in BLOCK 1. (**b**, **c**) Significant power peaks (centered on 7–8 Hz) appeared in BLOCK 2 and BLOCK 3. (**d**) Averaging the last two blocks (BLOCK 2 + 3) showed a progressive increase in the 7–8 Hz peak with increasing STAI-T score. The smooth curves are the fitted polynomial functions based on the significant polynomial component detected by ANOVA.

We calculated GCSR, by taking the difference in EEG power between stop and go trials for short, medium, and long Stop Signal Delays and then subtracting the average power for short and long from medium (see GCSR calculation section in “Methods” section). We had previously found variation in GCSR across the three testing blocks of the SST with the frequency-power curve for block 2 not being intermediate between block 1 and block 3. An initial analysis of the current student data (Fig. 2a–c) again found significant variation of the frequency-power function across blocks with a relatively narrow power peak centered on 7–8 Hz appearing in block 2 in the high STAI-T (T > 45) group and in block 3 in the medium STAI-T group (T = 36–40) with the low STAI-T group (T < 33) trending to an inverted peak in block 3 (STAI-T × block[quad] × frequency[order 4], F_2,45_ = 6.482, *P* = 0.003). To explore the source of this interaction, a post hoc ANOVA was run on block 1 alone (Fig. 2a) and found no significant effects (Block 1 only, STAI-T × frequency, all F < 1.9, all *P* > 0.15). Treating this as an initial ‘training’ run, for all subsequent ‘testing’ analyses, we therefore excluded block 1 and analyzed data averaged over Block 2 and Block 3.

In students, as shown in Fig. 2d, high ‘theta’ corresponded with high STAI-T scores (~ 5–9 Hz, peak [0.114 Log µV^2^] ~ 7 Hz), low ‘theta’ with medium STAI-T scores (~ 6–10 Hz, peak [0.037 Log µV^2^] ~ 8 Hz), and ‘theta’ was absent (− 0.037 at 7 Hz) with low STAI-T scores (STAI level × frequency[order 6], F_2,45_ = 4.16; *P* = 0.022). The difference between groups with high and low STAI-T scores reversed above 10 Hz.

**Figure 3:**
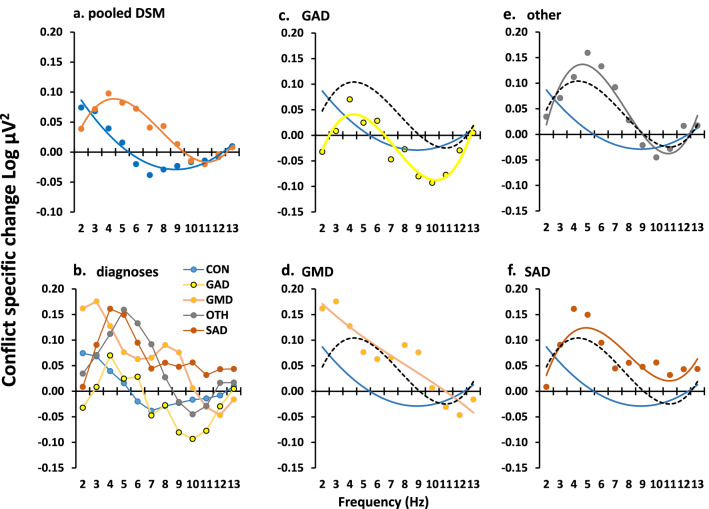
Variation of GCSR in the community sample. (**a**) Patients, pooled across diagnosis, showed a peak in the region of 4–5 Hz. However, the difference curve (Fig. 4b) shows that they differed maximally from controls in the region of 6 Hz and the asymmetry of the peak within the analysed range gave rise to the cubic component reported in the main text. Post hoc analysis restricted to 3–10 Hz, to achieve symmetry, resulted in the expected significant quadratic component (compare with 2SE quadratic shown in Fig. 4b). (**b**) Diagnostic groups overlayed with no trend lines. (**c**–**f**) Individual diagnoses with overlaid trendline and similar trendlines for control (blue) and average of diagnosis averages (black, dashed) for comparison. Abbreviations: *CON* control, *GAD* generalized anxiety disorder, *GMD* generalized anxiety with major depression, *OTH* other anxiety diagnoses (e.g., panic disorder), *SAD* social anxiety disorder.

**Figure 4:**
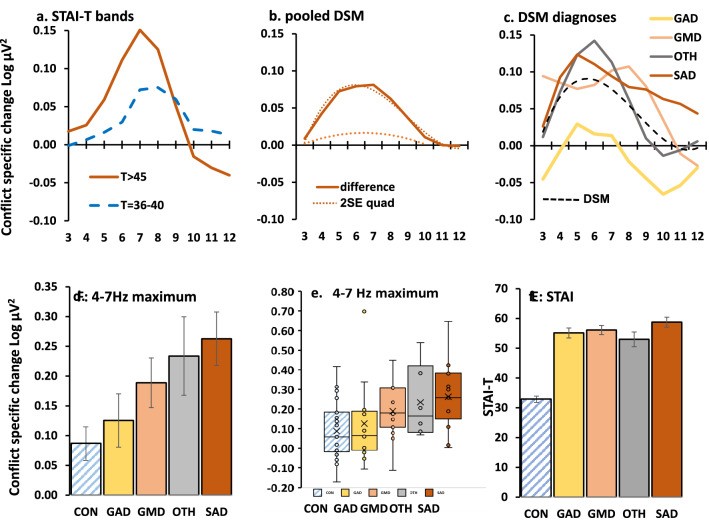
Clinically relevant differences from control in goal conflict specific EEG power (GCSR, 3-point smoothed after ANOVA) at the right frontal site F8 (F7 for left handers). (**a**) Power difference for clinical level (T > 45, high GCSR) and subclinical (T = 36–40, modest GCSR) relative to low (T < 33) STAI-T groups. (**b**) high GCSR in patients relative to controls (largest difference at 6–7 Hz) with (dotted) fitted quadratic and cubic curve detected by ANOVA. The lower dotted curve is 2SE for the quadratic difference in the range 3–10 Hz. (**c**) Variation in difference from controls with diagnosis. Removal of the control group eliminated significant differences from the ANOVA, with the diagnostic groups sharing a significant cubic trend (dashed line) reflecting a common tendency to peak in the region of 5–6 Hz. (**d**) Variation across diagnostic groups in group average of individual maximum GCSR in the range 4–7 Hz. The apparent variation in maximum GCSR was not significant after removal of the control group. Bars represent 2SE. (**e**) Distribution of maximum 4–7 Hz GCSR scores across diagnostic groups. (**f**) STAI-T did not vary among the clinical diagnoses. Abbreviations: *CON* control, *DSM* American Psychiatric Association’s Diagnostic and Statistical Manual-IV, *GAD* generalized anxiety disorder, *GMD* generalized anxiety with major depression, *OTH* other anxiety diagnoses (e.g., panic disorder), *SAD* social anxiety disorder, *STAI* Spielberger State Trait Anxiety Inventory, *T* trait score on STAI.

Patients (combined over all diagnoses) differed from their controls with a frequency-power difference curve similar to that for of the high versus low STAI-T student groups (Fig. 3a; group × frequency[quadratic], F_1,85_ = 6.239, *P* = 0.014; group × frequency[cubic], F_1,85_ = 3.828, *P* = 0.054). Given the cross over at 10 Hz (as in the student data), we undertook a single post hoc ANOVA limited to 2–10 Hz. This resulted in a simple U-shaped *difference* (see Fig. 4b for 2SE quadratic difference; group × frequency[quadratic], F_1,85_ = 9.741, *P* = 0.002) that was maximal between 5 and 7 Hz. While the *difference* function (Fig. 4b) is very similar to both the student data (and the original drug difference, also obtained in students), the background curve in both groups of this older community population shows higher power in the 3–5 Hz range (frequency[linear], F_1,85_ = 8.170, *P* = 0.005; group × frequency[linear], F_1,85_ = 0.136, *P* = 0.714).

Analysis of the DSM diagnostic groups retained the effect of diagnosis (Fig. 3b; DSM × frequency[cubic], F_4,75_ = 2.859, *P* = 0.029). After exclusion of the control group there was a highly significant overall cubic trend resulting from a peak in the 5 Hz region with a reduction to zero in the 10–12 Hz region (Fig. 4c, dashed curve, frequency[cubic], F_1,43_ = 12.191, *P* = 0.001). There was no significant difference between DSM diagnoses (DSM × frequency[cubic], F_3,43_ = 2.068, NS; see Fig. 3c–f for individual fitted functions). A similar analysis of STAI scores (Fig. 4f) found a strong effect when controls were included (DSM, F_4,75_ = 72.475, *P* < 0.0001) as would be expected given our exclusion procedure for controls and patients; but there was no difference between diagnoses when controls were excluded (DSM, F_3,75_ = 1.289, NS).

Given the apparent variability in peak frequency across diagnostic groups and individuals, for distributional analysis we extracted the maximum power value for each individual in the 4–7 Hz range. This differed with diagnosis (Fig. 4d): control < generalized anxiety < comorbid generalized anxiety and depression < mixed other diagnoses < social anxiety (mean [95%CI] in Log µV^2^ = 0.087 [0.031–0.143]; 0.125 [0.036–0.215]; 0.189 [0.106–0.272]; 0.234 [0.102–0.365]; 0.263 [0.174–0.352]—respectively); which was significant when controls were included (DSM, F_4,75_ = 3.53, *P* = 0.011) but not when they were excluded (DSM, F_3,43_ = 1.416, NS). As predicted, high ‘theta’ was not specific to any particular diagnosis (Fig. 4e); and, even with social anxiety (which had the highest mean), only half the cases were in the top quartile for all participants.

## Discussion

As predicted (Fig. 1) by the Behavioral Inhibition System theory^17,19–21^, goal conflict-specific EEG ‘theta’ (4–12 Hz) rhythmicity^39^ is elevated in at least some cases in conventionally-recruited students selected for clinical levels of trait anxiety (Fig. 4a). The pattern of *change* shown by this ‘training sample’ was then also found in community anxiety cases confirmed by MINI diagnosis (Fig. 4b). In the patient cohort, this GCSR elevation cuts across conventional diagnoses (with some apparent distributional variation (Fig. 4c). Importantly, the variation of GCSR between current diagnostic categories contrasts with the similarity of their STAI-T scores (Fig. 4f). This suggests that GCSR has special diagnostic power that STAI-T lacks. GCSR could, therefore, be used as a biomarker for an anxiety process underlying a previously undefined psychiatric disorder at the group level. The results also strengthen the hypothesis that ‘theta’ measured in single dose experiments in rats^25^ or healthy humans^38,39^ could be used to predict the clinical effectiveness of novel anxiolytic drugs when delivered long-term.

We emphasize that we do not think the process detected by our prospective biomarker is sufficient for the disorder specifically related to it; nor is it necessary for what DSM or ICD currently group together as classes of “anxiety”^15^ disorder. We argue^43^ that there is a general class of neurotic disorders^44^, where a “double hit” involving two distinct personality traits is necessary for the disorder to manifest: one, more specific, trait determines which type of neurotic disorder will occur; and a second, more general, trait is both a risk factor for, and a necessary factor underlying, neurotic disorders in general. In the case of the trait detected by our biomarker, we would expect both it (indexed by ‘theta’) and the general trait to be high; and if either of these is reduced—by an anxiolytic or ketamine^45–47^, respectively—then the disorder will be ameliorated (slowly or quickly, respectively). This would account for nominally healthy cases (in the sense of those student recruits with an STAI < 45) that have high goal conflict ‘theta’ (Fig. 4e). We also argue^43^ that there are a range of anxiolytic-insensitive neurotic disorders where the “second hit” is, for example, high periaqueductal gray reactivity in those diagnosed with panic disorder^48,49^. Critically, with a neurotic background, ‘theta’ pathology could evoke panic attacks as a symptom, and panic pathology could elicit (via learning) otherwise normal ‘theta’ at an undesirably high level, or both could co-occur. Thus, as noted more generally in the introduction, “anxiety” and “panic” symptoms (and so varying diagnoses across Fig. 4e) could co-occur with either or both (comorbid) pathologies^13^.

Our results provide a form of proof-of-concept for identification of the neural basis of a particular class of mental disorder; and so a basis for developing a diagnostic entity. We are currently testing with fMRI whether the GCSR generated in the SST is linked to hippocampal activation (that then activates frontal cortex) or to purely frontal activation by goal conflict. However, the current measure was designed for maximum theoretical specificity and validity under research conditions. It needs enhanced sensitivity and stability if it, or a derived measure, is to be used in the clinic.

One possible way to improve our measure (based on a simple, theoretically-derived, linear × quadratic contrast at a single electrode site) is to use machine learning. We have already demonstrated that a convolutional neural net that adjusts both the weights of the contrast, and electrodes included in the calculations, can predict STAI scores with 4 times greater variance accounted for than our current measure^50^. However, to determine the basis of the network solution requires much more data and deconstruction of the network solution. It would also require drug validation of each of the discovered network components as it remains to be demonstrated that its increased prediction is via more sensitive detection of a specific anxiolytic-related process and not via, for example, an addition of detection of depression (which links to high STAI scores), which is pharmacologically distinct.

A second way would be to alter the task platform. The SST does not include explicit positive or negative reinforcers—so its generation of goal conflict must be weak. Novel human work with virtual predators^51,52^ finds regional patterns of activation using fMRI consistent with^53^ the neurology of BIS theory and anxiolytic-sensitive behavioral effects^54,55^. EEG recording in these novel tasks should produce similar goal conflict ‘theta’ responses to those obtained in our SST but at much higher motivational levels. However, the relevant measures derived from these tasks require drug validation to be tightly linked to the BIS theory and are likely to need avoidance-avoidance^54,55^ rather than approach-avoidance conflict to avoid the problems of ensuring control of motivation levels in clinical populations.

A previous metanalytic review has linked conventional (i.e. 4–8 Hz) frontal midline EEG theta power with anxiety and anxiolytic action^56^. This response differs from GCSR not only in its frequency and midline location (and so source likely in rostral anterior cingulate cortex^56^ rather than right inferior frontal gyrus^40^) but also because the main focus of the work (particularly with anxiolytic drugs) was on evoked potentials not rhythmicity and on outcome conflict (i.e., a period of post-response error detection) not goal conflict (i.e., a period of conflict between upcoming choices). The work with anxiolytic drugs also used only classical anxiolytics such as alcohol or lorazepam^57–59^ and did not make an explicit comparison with buspirone, which shares only anxiolytic action and not side effects^38–40^. This particular frontal midline response also contrasts with work on bursts of frontal midline theta rhythmicity that has an opposite relation to neuroticism and anxiety, and which is increased by both classical anxiolytics and, importantly, buspirone^24^. High power 4–8 Hz frontal midline theta has also been seen during the period *prior* to risky choices, correlating with trait anxiety, and reduced by the wearing a crash helmet during task performance^60,61^. However, like the outcome-related studies this response has not been challenged with buspirone (which affects anxiety but not panic) and does not use an analytical contrast of the type with which we separate specific effects of goal conflict from simple anticipatory aversion.

Anxiolytic-sensitive right frontal ‘theta’ rhythmicity, derived from the neuropsychological theory of the Behavioral Inhibition System, appears to be a biomarker for a specific dysfunction of anxiety that cuts across symptom-based diagnoses. Goal conflict-specific EEG ‘theta’ (4–12 Hz) rhythm provides the first theoretically-derived biomarker for this, or any other, psychiatric disorder.

## Methods

### Participants

There were two distinct pools of participants that can be viewed as a ‘training’ and ‘testing’ sample, respectively: ‘students’ and ‘community’ (see Supplementary Methods for full details of recruitment). Community recruits included ‘patients’ (self-identifying as suffering from anxiety, confirmed by MINI DSM-IV diagnosis—see Supplementary Methods), and healthy individuals who volunteered “for a research study into the links between specific personality traits and specific patterns of rhythmic brain activity” to match the patients’ demographics. Students (total N = 79) with STAI-T scores of 46–61, in a range typical of anxiety disorder patients^62^, were placed in a ‘high’ group (♀ = 13; ♂ = 4) with ‘medium’ (36–40; ♀ = 13; ♂ = 4) and low (24–32; ♀ = 13; ♂ = 4) groups gender matched to them by excluding cases at the boundaries between groups rather than within them (N = 10, STAI = 41–45; N = 17, STAI = 33–35; N = 1, STAI = 23). Note that the primary aim here was gender matching with retention of homogeneity within groups and separation between groups to match the ANOVA approach taken. The community groups had overlapping STAI-T scores and so 7 patients (STAI-T < 44) and 6 controls (STAI-T > 44) were removed from primary analysis, delivering final N = 33 (4 left-handed, based on self-report and mouse use) and N = 47 (5 left-handed), respectively. For additional analysis patients were subdivided by interview-confirmed (see Mini International Neuropsychiatric Interview in Supplementary Methods) diagnosis: GAD = generalized anxiety disorder; GMD = generalized anxiety with major depression; OTH = other anxiety diagnoses (e.g., panic disorder); SAD = social anxiety disorder. The study protocol was approved by the University of Otago Ethics Committee (Health: H15/005), and all participants provided written informed consent before taking part in the experiment. The authors assert that all procedures contributing to this work comply with the ethical standards of the relevant national and institutional committees on human experimentation and with the Helsinki Declaration of 1975, as revised in 2008. For details see Supplementary Methods.

### Procedures

Questionnaires and a stop-signal task (SST, for full details see Stop Signal Task in Supplementary Methods) were presented on a PC computer screen using the same procedures as our previous experiments^39^.

Responses to personality questionnaires were collected for future analysis of the correlations between current measures of personality and EEG. Only the STAI-T was used to differentiate participants in the current analyses. For this reason, other personality measures (see Questionnaires in Supplementary Methods) are not reported here.

EEG was recorded with standard procedures (see EEG recording in Supplementary Methods) with bandpass filters set at 1–36 Hz, and down-sampled to 128 Hz for analysis. Only the right frontal site, F8, is reported here as previously for right handers^39^, with the left frontal site F7 being substituted for left handers. We have recently shown^63^ that in, demographically matched groups, left-handers’ GCSR power distribution is largely the mirror image of right handers’; and that there are no significant differences between left-handers’ F7 and right handers’ F8.

### Stop signal task (SST)

**Figure 5:**
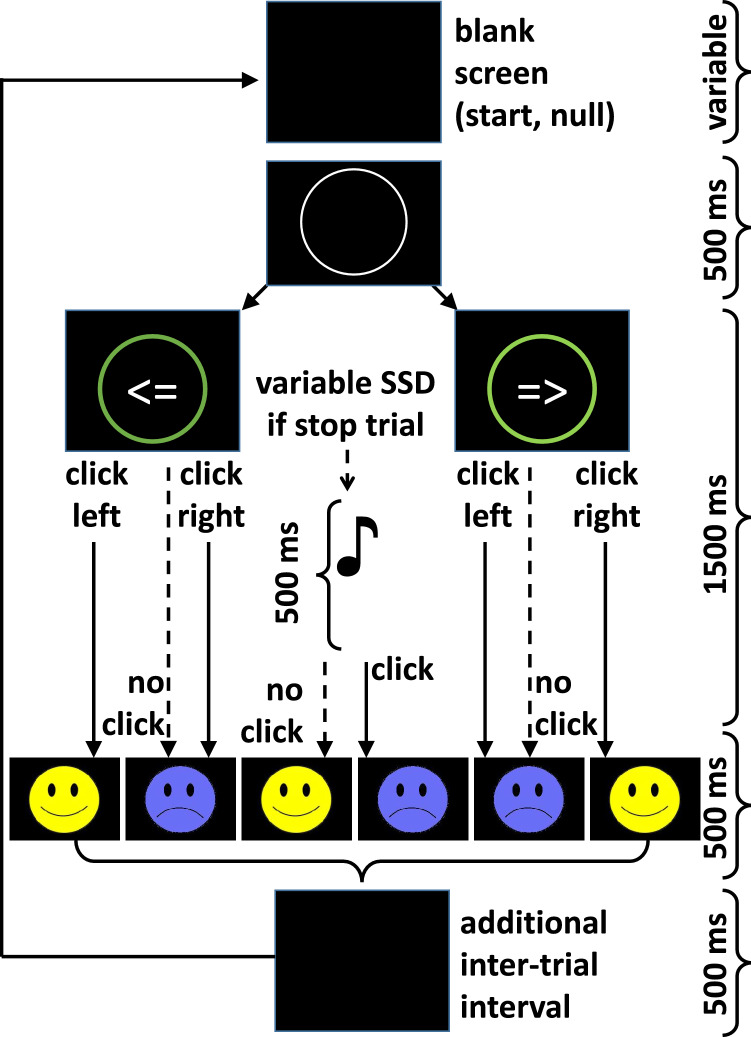
Sequence of events in the stop-signal task. Each trial started with a white fixation circle appearing in the middle of a blank screen. This circle then turned green when the Go signal (left/right arrow) appeared inside. In some trials this was followed by a stop signal (1000 Hz tone) being presented at varied stop signal delays (SSD). Feedback on performance was delivered in the form of a smiley or frowny face depending on the participant’s response. Adapted from^39^.

For the SST (see Fig. 5), right-handed participants placed their index finger on the left button, and their middle finger on the right button to respond to the corresponding left/right arrows on the screen. Left-handed participants placed their middle finger on the left mouse button and their index finger on the right mouse button to make left/right arrow responses. Participants were asked to respond as fast as possible to stimuli appearing on the screen using the computer mouse. On trials where there was an auditory tone, participants were instructed to try and inhibit their response. The importance of responding as fast as possible on both go and stop trials was emphasized by the experimenter.

Our SST had three blocks of trials, each separated by a one- to two-minute rest break. Each block contained 132 trials, comprising of 99 Go trials and 33 Stop trials. Trials were pseudo-randomized with 1 Stop trial and 3 Go trials in each set of 4 trials. The Stop trials were programmed to occur in different positions during each set of 4 trials, but the pattern of presentation was identical for every participant.

An important feature of our version of the SST^39^ is that the stop signal delay varies within three bands short, medium, and long—each adjusted to the participant’s ongoing behavior. This generates 11 of each of three types of trials where stopping is difficult (~ 25% correct), easy (~ 75% correct), or stopping and going are in balanced conflict (~ 50% correct). This provides the basis for the conflict-specific contrast used in analysis.

### Data processing

Behavioral data were processed as usual^39^ (see also Supplementary Methods), but are not reported here as they are not sensitive to anxiolytic drugs and do not correlate with our biomarker and so are not relevant to the current analysis.

Electrophysiological data were also processed as previously^39^. After removing artefacts from the recordings (see Artefact Removal in Supplementary Methods), a 1-s Hanning window was applied to each trial. For Stop trials, the 1-s Hanning window was applied 0.25 s before the presentation of the stop signal (auditory tone) through to 0.25 s after the stop signal had ended. On the Go trials, the Hanning window was located similarly, based on where the stop signal was delivered in the adjacent Stop trial. The cosine wave function of the Hanning window extracts most power during the middle 0.5 s and the least during the leading and trailing 0.25 s. It improves frequency resolution in the subsequent Fourier transform twofold in comparison to a 0.5 s square window as well as improving the quality of the transform. A Fourier transform was then applied and converted to the power spectrum, which was log transformed to normalize error variance before Stop/Go trials were averaged for each participant.

Stop trials were averaged for each stop signal delay (SSD) type for each of the three testing blocks, as were their matching (adjacent) go trials. Where there were any missing data within a Hanning window, the entire spectrum for that trial was replaced with missing values. Where there were less than 7 trials without missing values, the average was replaced by missing values.

### Statistical analysis

#### GCSR calculation

GCSR for each participant was computed as a nominal linear (stop, go) × quadratic (short, medium, long SSD) orthogonal polynomial contrast^64^. The average Go power was subtracted from the average Stop power for each SSD type to extract power specific to Stopping. Then the average of short and long SSD stop-specific power was subtracted from the medium SSD stop-specific power to extract power specific to goal conflict. Maximum conflict was expected to occur to the stop signal with the medium SSD, as Going and Stopping are equally likely during this condition. In contrast, low levels of conflict were expected to occur during short and long SSDs, with other factors (such as percent correct: short =  ~ 25%; long =  ~ 75%) tending to average to the value expected for medium SSDs (~ 50%). Note that the F ratios obtained by ANOVA of these explicit GCSR values are identical to those that would be obtained for the Trial type [linear] × SSD[quadratic] interaction with the original data.

#### Smoothing

As an improvement on our previous methods, a 3-point running mean across frequencies was used to smooth each participant’s GCSR to reduce the jitter of power between adjacent frequencies inherent in the Fourier Transform. This smoothing narrows the frequency band by one data point at each end, reducing an initially selected 1–14 Hz to our 2–13 Hz band of interest (based on our expectation that power peaks would occur in the 4–11 Hz range). Since the primary statistical tests were of orthogonal polynomial trends (see below), we also smoothed the means with a second 3-point running mean for Fig. 4. The unsmoothed means and trend decompositions are presented in the other figures that analyse the significant trends in more detail.

#### Analysis of variance

Analysis of GCSR was restricted to the F8 channel (F7 in left-handers), as this is the only location where correlations between GCSR and trait anxiety were previously found in the SST^35^ and is also the site where we obtained our clearest previous results^37–39^ Analysis of Variance (ANOVA) was computed using the IBM SPSS Statistics Package 25 (IBM North America, New York, NY, USA). Factors included in this analysis were frequency (2–13 Hz), block (1–3), and groups. For student participants, groups had 3 levels (high, medium, low STAI-T); for the main patient analysis, groups had 2 levels (patients, controls); and for analysis of DSM-IV diagnoses, groups had 5 levels (control, GAD, GAD with concurrent MDD, SAD, and other). Frequency and blocks were automatically assessed for orthogonal polynomial components by SPSS.

The experimental task, the choice of only F8 for analysis (F7 for left handers), and the focus on the stop–go[linear] × SSD[quadratic contrast] to derive GCSR are all based on our previous work^38–40,63^. Figure 1 is a re-analysis for the block2 + 3 average of previously reported^39^ drug data—shown inverted to generate a curve representing the expected effect of high STAI. The choice of blocks to be analysed for the community sample (average of last two rather than trends across all three) is based on a single initial analysis of the student data. This choice was made both to increase the simplicity of the presented data and because, where a simple trend is present, analysis of the endpoint can be at least as, and often more, informative than analysis of all the data points across the trend. Note that in all cases there is an a priori prediction as to the direction (and nature) of the differences. Post-hoc testing involved polynomial functions of frequency with only 1 df and did not test individual frequencies separately. For these reasons, the tests have been applied without Bonferroni correction of the significance values. Likewise, the DSM group analysis assumes that some diagnostic groups will show this same previously predicted effect (which since they are subgroups from the original community analysis is close to a mathematical necessity) but leaves open only the question of which will deviate from which, if any, and whether any will be like control.

## Supplementary Information

Supplementary Information.

## Data Availability

The data that support the findings of this study are available from the corresponding author upon reasonable request.
