# Supplementary material for: Right frontal anxiolytic-sensitive EEG ‘theta’ rhythm in the stop-signal task is a theory-based anxiety disorder biomarker
Source: Sci Rep. 2021 Oct 5;11:19746. doi: 10.1038/s41598-021-99374-x (PMC8492763; doi:10.1038/s41598-021-99374-x)
Supplement: Supplementary file 1 — Supplementary Information. [file 41598_2021_99374_MOESM1_ESM.pdf]

Right frontal anxiolytic-sensitive EEG 'theta' rhythm in the stop-signal task is a theory-based anxiety disorder biomarker

SUPPLEMENTARY METHODS

Shabah M. Shadli[1] PhD, Lynne C. Ando[1] BSc, Julia McIntosh[1] MSc, Veema Lodhia[4] PhD, Bruce R. Russell[2] PhD, Ian J. Kirk[4] PhD, Paul Glue[3] MD FRCPsych and Neil McNaughton[1*] PhD

Affiliations:

*[1]Dept. Psychology, [2]School of Pharmacy, and [3]Dept. Psychological Medicine, University of Otago, Dunedin, New Zealand. [4]Dept. Psychology, University of Auckland, Auckland, New Zealand. *Corresponding author.*

ORCID IDs: SMS = 0000-0002-3607-3469; VL = 0000-0002-3205-9811; BRR = 0000-0001-6935-1974; IJK = 0000-0002-0444-6718; PG = 0000-0002-7305-2800; NMcN= 0000-0003-4348-8221

Correspondence to: Professor Neil McNaughton
Department of Psychology
University of Otago
PO Box 56
Dunedin 9054
New Zealand

Phone: 64 3 479 5835; **Fax:** 64 3 479 8335; **Email**: neil.mcnaughton@ otago.ac.nz

Running title: Anxiety disorder biomarker

Funding: Health Research Council of New Zealand

Contents

Supplementary Methods and Materials ***3***

Participant Details **3**

Subgroup division **4**

Mini International Neuropsychiatric Interview (MINI) **5**

Questionnaires **5**

EEG Recording **5**

Low density recording 5

High Density recording 6

Stop Signal Task (SST) **7**

SST control flow 7

Stop Signal Delay Staircases 8

Data Processing **9**

Behavioural Data 9

Artefact Removal 9

Supplementary References ***10***

Supplementary Methods and Materials

Participant Details

There were two distinct pools of participants: ‘students’ and ‘community’.

Students and community controls: Participants in the student group were recruited from Otago Student Job Search (SJS), an agency through which students obtain casual work, including acting as experimental participants. They all reported being right-handed. Participants in the healthy control group were recruited through online advertisements on Facebook “for a research study into the links between specific personality traits and specific patterns of rhythmic brain activity” to match the patients’ demographics. All students and community controls reported no major illness in the past month, were not prescribed any psychoactive medication in the previous six months, and stated that they had not consumed alcohol in the 24 hours prior to participating in the study. None disclosed having been treated for anxiety, depression or other mood disorders in the previous 12 months. Monetary reimbursement (above the minimum wage rate at the time) was given to participants in appreciation for their time and effort.

Patients: Participants in the community patient group were recruited from public health organizations in Dunedin and Auckland and using advertisements within local newspapers, supermarkets, and Facebook. This group consisted of individuals who reported experiencing chronic symptoms of anxiety or fear, but were not receiving any pharmacological treatments for relief. Medication-free patients were selected for this sample as anxiolytics can alter the strength of GCSR signals [1]. Participants were excluded if they had a history of substance abuse or neurological disorder. Patients’ diagnoses were confirmed with a MINI diagnostic examination (see below). Similar to the control group, these participants were otherwise healthy and reported no significant illness in the past month, no use of psychoactive medications in the past six months, and no alcohol consumption in the 24 hours before the experiment. All patient participants received vouchers (NZ$30 worth) as compensation for their time and travel.

Subgroup division

Participants recruited from SJS were separated into demographically matched groups of high, medium and low STAI-T scorers for an initial ANOVA to investigate the relationship between STAI-T and GCSR. The initial cut-off STAI-T score used in the present study to separate SJS participants into the high group was taken from a review of six treatment outcome studies [2]. The mean STAI-trait values obtained from clinical GAD groups ranged from 47 to 61 across the studies. The review concluded that an STAI-T of 46 is the cut-off point for clinically significant anxiety, with those above this being functionally impaired. We found 17 individuals above this cut off. We then gender matched (♀= 13; ♂= 4) two lower scoring groups to them by excluding cases at the boundaries between the groups (N=10, STAI = 41-45; N=17, STAI = 33-35; N=1, STAI = 23). The resultant STAI-T score ranges were: low = 24-32; medium = 36-40; and high = 46-61.

Community participants were analysed as two groups, “healthy control” and “patient”, initially to assess the simple effect of patient status. The community groups had overlapping STAI-T scores and so 7 patients (STAI-T<44) and 6 controls (STAI-T>44) were removed from primary analysis. This resulted in final N= 33 (4 left-handed, based on self-report and mouse use) and N = 47 (5 left-handed), respectively. For a later analysis of diagnostic differences, patients were separated into four groups based on their MINI DSM-IV diagnosis that were intended to maximise the capacity to detect diagnostic differences while also balancing group size. We, therefore, split the large GAD group into subgroups with and without MDD; and, conversely, we pooled a range of infrequent diagnoses into ‘others’. The resultant groups were: 1) GAD alone (N=13); 2) GAD with concurrent MDD (N=15); 3) SAD (N=13); and, 4) others (patients with primary diagnoses of panic, anorexia, PTSD etc.; N=6).

Mini International Neuropsychiatric Interview (MINI)

As part of the recruitment procedure, participants in the patient group were administered the MINI ver 6.0.0; [3]. The MINI is a short structured interview created to assist diagnosis of common Axis-1 psychiatric disorders presented in DSM-IV and ICD-10 [4]. Despite its brevity, the MINI is reported to have similar levels of validity and reliability to other structured interviews used in the clinic [5]. Interviews for this study were carried out by a psychiatrist, or a clinical psychology trainee trained by the psychiatrist, in a quiet, secluded room.

Questionnaires

Demographic data relating to gender, age, ethnicity, and handedness were obtained from all participants before running the questionnaires. This information was collected using the standard Statistics New Zealand format. Participants were required to complete two sets of personality questionnaires. The first set of questionnaires were presented before cap fitting. It included the Trait Anxiety scale items from the STAI State-Trait Anxiety Inventory; [6], the Extraversion and Neuroticism items from the EPQ-R Eysenck Personality Questionnaire-Revised; [7], and the BIS items from the BIS/BAS scale Behavioural Inhibition System/Behavioural Activation System; [8].

The second set of questionnaires was delivered after the participant had finished the SST. This set consisted of ten scales from the PID-5 Personality Inventory for DSM-5; [9] and included the Depressiveness, Anxiousness, Emotional Liability, Perseveration, Separation Insecurity, Withdrawal, Anhedonia, Risk-taking, Intimacy Avoidance, and Restricted Affectivity scale items. In addition, probes regarding sleep and history of depression were included within this series.

EEG Recording

Low density recording

The bulk of the data were collected with low density recording in Dunedin. Waveguard caps assembled with Ag/AgCl electrodes, connected to 32 channels of an ASA Neurotechnology system (ANT Neuro, Enschede, The Netherlands) were used for recording. Participants were fitted with a small (48-53cm), medium (53-57cm) or large (57-62cm) EEG cap depending on the circumference of their head. EEG signals were recorded, with CPz as recording reference and electrode F8 of the

International 10:20 system used for GCSR amplitude analysis. Ocular artefacts caused by eye-blinks were detected via Fp1, and GND (anterior to Fz) was used as the ground electrode. All electrodes were later re-referenced to the common average of M1 and M2 mastoid electrodes. Impedance at each electrode was lowered to below 20kΩ by injecting conductance gel, One Step Cleargel (H + H Medizinprodukte GbR, Münster, Germany) using a 10mL syringe with a 16-gauge rounded needle (Precision Glide Needle, Becton Dickinson, Franklin lakes, NJ, USA) between the electrodes and the participant's scalp. Level of impedance at each electrode was displayed on the recording computer by the ASA program. Neural activity was recorded at a sampling rate of 512Hz, with bandpass filters set at 1-36 Hz, and down-sampled to 128Hz for analysis. All EEG recordings were carried out in a certified body protected room equipped with the apparatus described above.

High Density recording

Some community control and patient recordings were carried out in Auckland in an electrically shielded room (IAC Noise Lock Acoustic - Model 1375, Hampshire, United Kingdom) using 128 channel Ag/Agcl electrode nets (Electrical Geodesics Inc., Eugene, OR, USA) [10]. EEG was recorded continuously, sampling rate was 1000 Hz, and the analogue band pass was set to 0.1 to 400 Hz with Electrical Geodesics Inc. amplifiers (300-MΩ input impedance). The impedances were set below 40 KΩ which is the standard for this system [11]. Cz was the reference electrode. Vertical and horizontal electro-oculograms (VEOG and HEOG) were recorded and subsequently employed for artefact rejection. There were three different sizes of nets used based on the participant's head circumference. The net was soaked for 5 minutes in a solution containing 11g of KCl dissolved in 1L of warm water and 5 mL of baby shampoo. The net was gently applied to the head and if the impedance of any electrode was above 30 KΩ, more solution was applied under that electrode to decrease impedance.

Once appropriate levels of impedance were achieved, participants were asked to engage in jaw clenching and eye-blinking to check for any abnormalities in the signals and demonstrate to the participants the noise these movements create. Any visible anomalies were then adjusted by moving the cord or adjusting specific electrodes.

Stop Signal Task (SST)

Participants were then asked to complete the SST. Instructions for the task, as in Aron and Poldrack [12], were given verbally by the experimenter before starting. Participants were asked to respond as fast as possible to stimuli appearing on the screen using the computer mouse. On trials where there was an auditory tone, participants were instructed to try and inhibit their response. The importance of responding as fast as possible on both go and stop trials was emphasized by the experimenter during this explanation. After finishing the SST, participants were asked to complete the second set of questionnaires. Participants then had their caps removed, were cleaned of gel, thanked for their participation, and given their reimbursement before leaving the laboratory.

An important feature of our version of the SST [13] is that the stop signal delay varies within three bands short, medium, and long – each adjusted to the participant's ongoing behavior. This generates three types of trials where stopping is difficult (~25% correct), easy (~75% correct), or stopping and going are in balanced conflict (~50% correct). This provides the basis for the conflict-specific contrast used in analysis.

SST control flow

Figure 5 of the main text shows a flow diagram of the task. The basic task was as in Aron and Poldrack [12] with two main types of trial – Go and Stop. Trials were separated by a null time that ranged between 0.5 and 4 s (mean, 1 s; sampled from an exponential distribution truncated at 4 s) as in[12]. During the Go trials, a white fixation circle was presented in the middle of a black screen. A left (<=) or right (=>) arrow appeared inside this circle 500ms later, turning the fixation circle green. Participants were instructed to respond as fast as possible to the arrows using the corresponding buttons on the computer mouse (i.e., a right (=>) arrow required a right button click, left (<=) arrow a left click). The arrow and fixation circle were programmed to disappear after a response or following 1500ms of no response. During the Stop trials, a stop signal indicated by a 1000Hz tone was delivered at varying intervals (see next section). Participants were instructed to inhibit their responses on these trials by preventing themselves from clicking the mouse.

The current SST had three blocks of trials, each separated by a one- to two-minute rest break. Each block contained 132 trials, comprising of 99 Go trials and 33 Stop trials. Trials were pseudo-randomized with 1 Stop trial and 3 Go trials in each set of 4 trials. The Stop trials were programmed to occur in different positions during each set of 4 trials, but the pattern of presentation was identical for every participant.

There were a number of additions made to the original version of the task to improve its capacity to detect conflict-related effects, as detailed in Shadli, et al. [13]. As shown in Figure 2, feedback on performance was given to participants 500 ms after each response. On Go trials, participants received a smiley face for a correct response, a frowny face for an incorrect one, and a frowny face if they did not respond within 1500 ms after the arrow had been presented. A '*Slow!*' feedback was given to participants if they delayed their responses for too long. This was intended to prevent participants from attempting to increase their chances of stopping successfully later by strategically slowing down their Go response reaction times. On stop trials, a smiley face was presented if the participant successfully inhibited their response and a frowny face if they incorrectly made a response. Before the test blocks, participants completed a practice block consisting of 30 Go trials with no Stop trials. The practice block was a Choice Reaction Time (CRT) task similar to[14]. The purpose of the CRT was to stabilize and estimate the mean reaction time, which was then used to control delay values for the first Stop trials (see below).

Stop Signal Delay Staircases

The interval from the start of the trial to the stop signal (the stop-signal delay; SSD) varied across trials under control of a staircase tracking algorithm. In our task, similar to Carter, et al. [14], the short and long SSDs were programmed to be 20% and 80% of the mean reaction time for the previous 16 Go trials. For the medium staircase, the initial delay was set to 45% of the previous mean Go reaction times; but from that point on within the block the value then (as in all staircases of the original SST) changed across trials depending on the participant's performance on the previous Stop trial. If the participant was successful at stopping, the SSD value increased by 30 ms (not 50 ms[12]), making inhibition more difficult in the following Stop trial, and if they were unsuccessful the value decreased

by 30 ms, making inhibition easier in the subsequent trial. The medium SSD could also not move to within 50 ms of the SSD of the other staircases. It was expected that this procedure would track 50% correct stopping for intermediate trials and so produce maximum conflict between going and stopping; while having a clear separation between the SSDs of the three staircases.

Data Processing

Behavioural Data

For each trial in the SST: the trial and block number, trial type (Go or Stop), SSD, reaction time (RT), staircase index (1-3), movement on the staircase, and type of response (Left/Right or Null) was recorded. Based on these data, three summary behavioural measures were calculated. This included: (1) the average Go reaction time (GoRT, ms) across all Go trials; (2) average SSD from the middle staircase; and (3) the Stop-Signal Reaction Time (SSRT). The SSRT was calculated in accordance to the Horse Race Model [15], with the average SSD from the intermediate staircase subtracted from the median GoRT to produce SSRT.

Artefact Removal

EEG recordings contain undesired artefacts [16,17]. To remove these artefacts, data were processed through a set of purpose-built software routines. Residual mains noise was removed using a three-point running mean – equivalent to a low pass filter with a cut-off at 43 Hz. Eye blink artefact components were extracted by applying a template to the ballistic components of each eye blink recorded at Fp1 and then removing this component from the signals in the other channels after scaling for each channel with a least squares linear regression to leave residual EEG [18]. Muscle and other large artefacts were automatically replaced with missing values. The recordings were then checked visually and any outstanding eye-blink or muscle artefacts were manually removed by the experimenter and replaced with missing values.

Supplementary References

1 McNaughton, N., Swart, C., Neo, P. S. H., Bates, V. & Glue, P. Anti-anxiety drugs reduce conflict-specific "theta" – a possible human anxiety-specific biomarker. *J. Affect. Disord.* **148**, 104-111, doi:10.1016/j.jad.2012.11.057i (2013).

2 Fisher, P. L. & Durham, R. C. Recovery rates in generalized anxiety disorder following psychological therapy: an analysis of clinically significant change in the STAI-T across outcome studies since 1990. *Psychol. Med.* **29**, 1425-1434 (1999).

3 American Psychiatric Association. *Diagnostic and Statistical Manual of Mental Disorders*. 4th edn, (1994).

4 Sheehan, D. V. *et al.* The Mini-International Neuropsychiatric Interview (M.I.N.I): The development and validation of a structured diagnostic psychiatric interview for DSM-IV and ICD-10. *The Journal of Clinical Psychiatry* **59**, 22-33 (1998).

5 Lecrubier, Y. *et al.* The Mini International Neuropsychiatric Interview (MINI). A short diagnostic structured interview: reliability and validity according to the CIDI. *European psychiatry* **12**, 224-231 (1997).

6 Spielberger, C. D., Gorsuch, R., Lushene, R. E., Vagg, P. R. & Jacobs, G. A. *Manual for the State-Trait Anxiety Inventory (Form Y1 – Y2).* Vol. IV (1983).

7 Eysenck, H. J. & Eysenck, S. B. G. *Manual of the Eysenck personality scales (EPS adult) : comprising the EPQ-revised (EPQ-R) (including addiction and criminality scales), EPQ-R short scale, impulsiveness (IVE) questionnaire (implsiveness/ venturesomeness/empathy).* (Hodder & Stoughton, 1991).

8 Carver, C. S. & White, T. L. Behavioral inhibition, behavioral activation, and affective responses to impending reward and punishment: The BIS/BAS Scales. *Journal of Personality and Social Psychology* **67**, 319-333, doi:10.1037/0022-3514.67.2.319 (1994).

9 American Psychiatric Association. in *Diagnostic and Statistical Manual of Mental Disorders* (2013).

10 Teyler, T. J. *et al.* Long-term potentiation of human visual evoked responses. . *Eur. J. Neurosci.* **21**, 2045-2050, doi:doi: 10.1111/j.1460-9568.2005.04007.x (2005).

11 Ferree, T. C., Luu, P., Russel, G. S. & Tucker, D. M. Scalp electrode impedence, infection risk, and EEG data quality. *Clin. Neurophysiol.* **112**, 536-544 (2001).

12 Aron, A. R. & Poldrack, R. A. Cortical and subcortical contributions to stop signal response inhibition: role of the subthalamic nucleus. *Journal of Neuroscience* **26**, 2424-2433 (2006).

13 Shadli, S. M., Glue, P., McIntosh, J. & McNaughton, N. An improved human anxiety process biomarker: characterization of frequency band, personality and pharmacology. *Translational Psychiatry* **5**, e699, doi:10.1038/tp.2015.188 (2015).

14 Carter, J. D. *et al.* Assessing inhibitory control: A revised approach to the stop signal task. *Journal of Attention Disorders* **6**, 153-161, doi:doi:10.1177/108705470300600402 (2003).

15 Logan, G. D., Cowan, W. B. & Davis, K. A. On the ability to inhibit simple and choice reaction-time responses - a model and a method. *J. Exp. Psychol. Hum. Percept. Perform.* **10**, 276-291 (1984).

16 Reddy, A. G. & Narava, S. Artifact removal from EEG signals. *International Journal of Computer Applications* **77** (2013).

17 Teplan, M. Fundamentals of EEG measurement. *Measurement science review* **2**, 1-11 (2002).

18 Zhang, S. *et al.* Removing eye blink artefacts from EEG—A single-channel physiology-based method. *Journal of Neuroscience Methods* **291**, 213-220, doi:http://dx.doi.org/10.1016/j.jneumeth.2017.08.031 (2017).
